# Supplementary figures and images for: Host-microbiome metabolism of a plant toxin in bees
Source: eLife. 2022 Dec 6;11:e82595. doi: 10.7554/eLife.82595 (PMC9897726; doi:10.7554/eLife.82595)

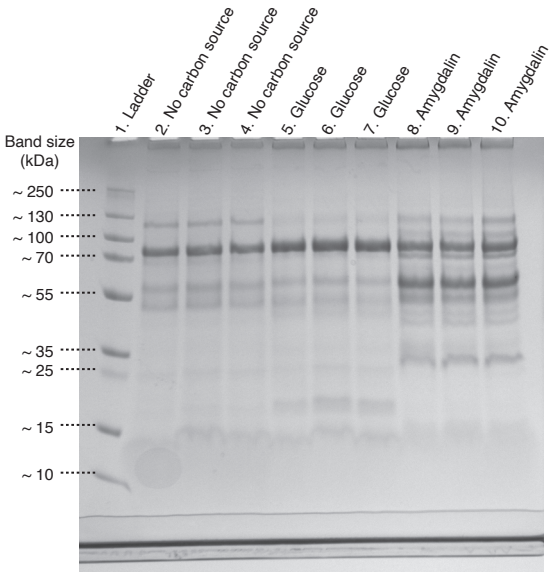

Supplement: Figure 4—source data 1. — From left to right, columns represent: (1) PageRuler Plus Prestained Protein Ladder; (2–10) Supernatants of cultures (1–4) grown in the absence of a carbon source, (5–7) in the presence of 10 mM glucose as sole carbon source, or (8–10) in the presence of 10 mM amygdalin as sole carbon source. Each sample (30 μL) was mixed with 5 μL of 6× SDS gel-loading buffer (0.35 M Tris-Cl pH 6.8, 10% w/v SDS, 0.012% w/v bromophenol blue, 30% v/v glycerol, 0.6 mM dithiothreitol), denatured at 100°C for 5 min, then run on a Bolt 4–12% Bis-Tris Plus, 1.0 mm, protein gel at 200 V for 22 min. [file elife-82595-fig4-data1.zip › Figure_4 - source data 1.pdf]

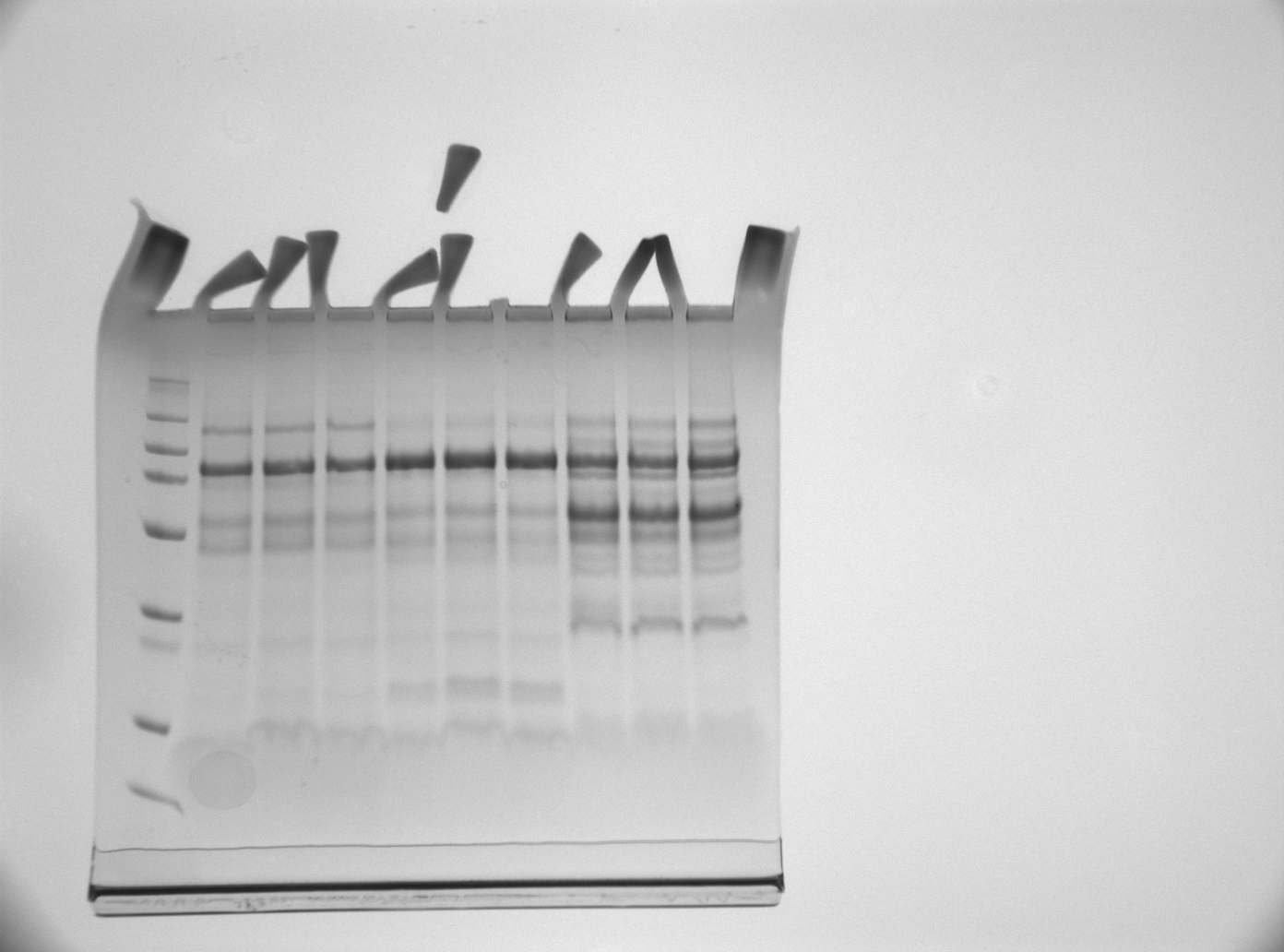

Supplement: Figure 4—source data 1. — From left to right, columns represent: (1) PageRuler Plus Prestained Protein Ladder; (2–10) Supernatants of cultures (1–4) grown in the absence of a carbon source, (5–7) in the presence of 10 mM glucose as sole carbon source, or (8–10) in the presence of 10 mM amygdalin as sole carbon source. Each sample (30 μL) was mixed with 5 μL of 6× SDS gel-loading buffer (0.35 M Tris-Cl pH 6.8, 10% w/v SDS, 0.012% w/v bromophenol blue, 30% v/v glycerol, 0.6 mM dithiothreitol), denatured at 100°C for 5 min, then run on a Bolt 4–12% Bis-Tris Plus, 1.0 mm, protein gel at 200 V for 22 min. [file elife-82595-fig4-data1.zip › Figure_4 - source data 2.tif]
